# The highs and lows of bird flight

**DOI:** 10.7554/eLife.50626

**Published:** 2019-09-03

**Authors:** Jon Harrison

**Affiliations:** School of Life SciencesArizona State UniversityTempeUnited States

**Keywords:** bar-headed goose, hypoxia, wind tunnel, high-altitude, metabolic rate, flight, Other

## Abstract

Bar-headed geese lower their flight metabolic rates to fly in low-oxygen conditions.

**Related research article** Meir JU, York JM, Chua B, Jardine W, Hawkes LA, Milsom WK. 2019. Reduced metabolism supports hypoxic flight in the high-flying bar-headed goose (*Anser indicus*). *eLife* **8**:e44986. doi: 10.7554/eLife.44986

Most animals live at low altitudes, where the atmosphere is rich in oxygen, and humans often gasp for air at higher elevations due to the reduced oxygen levels. High altitudes should pose greater problems for birds because flight is the most costly form of animal exercise and because the reduced air density at high altitude generates less lift during flight. The most incredible feat of bird flight is perhaps the bi-annual migration of bar-headed geese, which cross the Himalayas at altitudes usually between 5,000 and 6,000 meters. Moreover, mountaineers have reported seeing bar-headed geese flying at altitudes above 8,000 meters, where the partial pressure of oxygen is less than half of that at sea level.

The physiology of high-altitude avian flight has fascinated mountaineers and comparative physiologists for a century and, more recently, creative physiologists have begun to use new technologies to explore it in detail. We know that flying at sea level raises oxygen consumption rates by a factor of 10–15 relative to resting rates (Ward et al., 2002). The cardiovascular demands of trans-Himalayan flight are even higher, with the heart rates of bar-headed geese that migrate naturally rising linearly with altitude to reach almost 400 beats per minute above 5,000 meters (Bishop et al., 2015).

Birds have evolved many physiological adaptations to flight. For one, their ventilation rates are high despite low levels of carbon dioxide in the blood, suggesting that neural centers controlling ventilation are less inhibited by low carbon dioxide than in other animals. Birds also have large hearts, and large lungs that are thin and have high diffusing capacity, as well as muscles that are highly capillarized. These changes provide most birds with exceptional cardiovascular capacities relative to mammals.

Bar-headed geese exhibit these traits, and their physiologies are also specifically adapted to high-altitude flight (Scott et al., 2015). Compared to related species that fly at lower elevations, bar-headed geese have larger lungs, take deeper breaths and ventilate more (Scott and Milsom, 2007). Moreover, the transport of oxygen in the blood during hypoxia (oxygen levels lower than those at sea level) is enhanced by their hemoglobin, which has a higher affinity for oxygen than that of related low-altitude birds. They also have more capillaries in their muscles, and these capillaries are located near mitochondria (Scott and Milsom, 2006). To date, however, no one had studied the cardiovascular system of the bar-headed goose during hypoxic flight.

Now, in eLife, Jessica Meir, Julia York and colleagues – who are based at the NASA Johnson Space Center, the University of British Columbia, the University of Texas at Austin and the University of Exeter – report the first measurements of cardiorespiratory function for bar-headed geese in hypoxic flight (Meir et al., 2019). Meir et al. raised goslings so that they were imprinted on a human foster parent (either Meir or York), and taught them to fly alongside their foster parent (Figure 1). Next the geese were fitted with instruments to measure their heart rates and arterial and venous P_O2_ and made to fly in a wind tunnel mimicking the conditions of high-altitude flight. They also wore masks to measure their metabolic rates during flights where the oxygen levels were adjusted to correspond to sea level (21% O_2_) and altitudes of 5,500 (10.5% O_2_) or 9,000 meters (7% O_2_).

**Figure 1.**
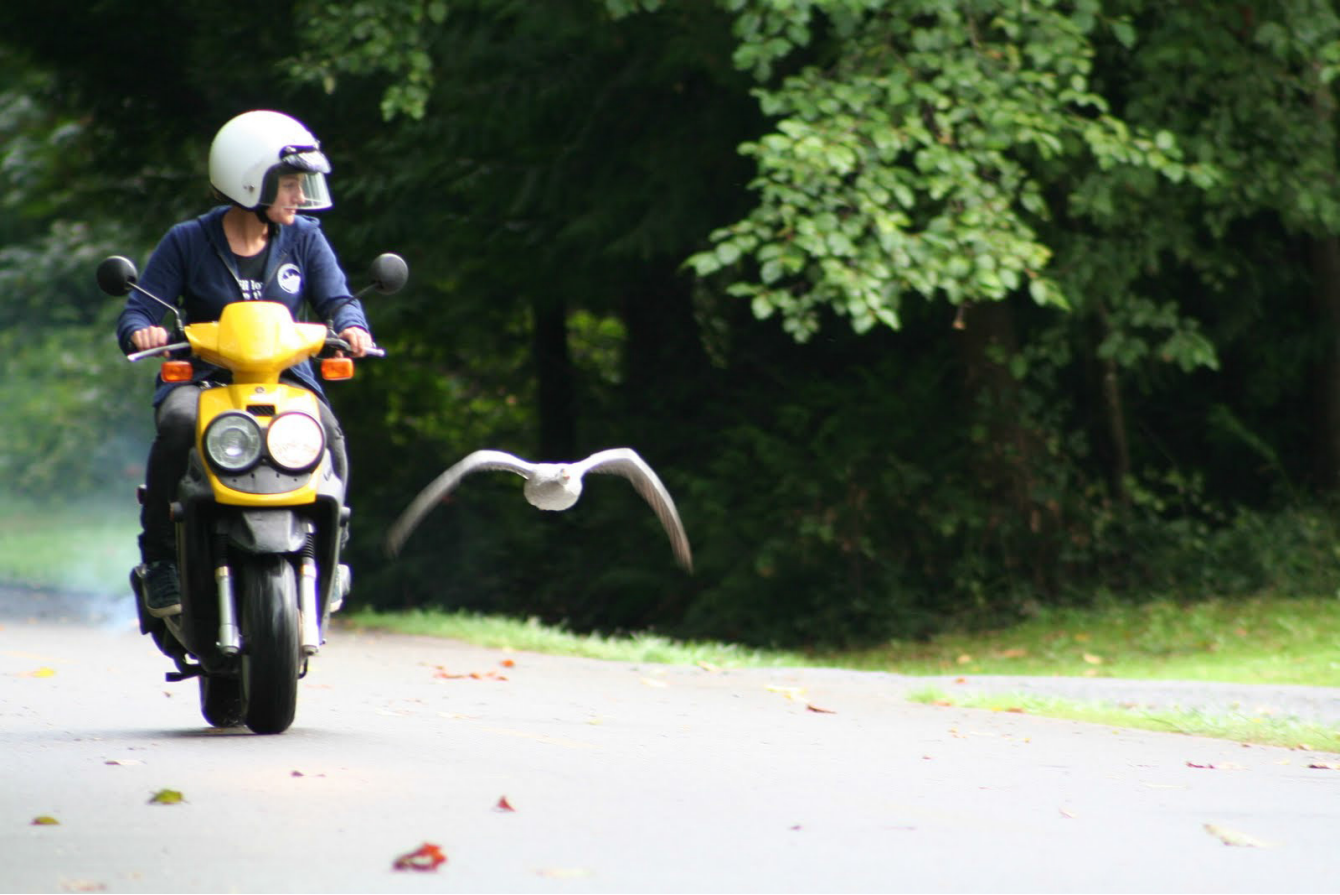
Teaching a bar-headed goose how to fly. The goslings used in experiments like these usually learn to fly in a wind tunnel. However, the wind tunnel that Meir et al. used in their experiments was broken when the first hatch of bar-headed geese fledged, so the researchers taught the geese to fly by riding alongside them on a bicycle and, later, a motor scooter.

Despite their well-demonstrated cardiovascular adaptations, flight in progressively more hypoxic air was associated with major decreases in arterial P_O2_ and moderate decreases in blood oxygen content. These changes likely led to a fall of 16–20% in average metabolic rate as the birds flew in hypoxia, but wing-beat frequency and heart rate did not change. These decreases in metabolic rate allowed the bar-headed geese to conserve energy, an important strategy for these migrating birds, but how can the geese continue to fly despite lower metabolic rates? Despite the decline in average metabolic rate in hypoxic flight, the minimum flight metabolic rate was unaffected by hypoxia. The timing of movements within each wing-beat changed during hypoxic flight, suggesting the geese may be moving their wings more efficiently. Alternatively, hypoxia may suppress the metabolism of tissues not involved in flight.

Another open question is what happens to the geese’s physiologies when they ascend in hypoxic air. Since the air is thinner, the birds will need more power, potentially leading to an inability to suppress their flight metabolism. Meir et al. showed that venous P_O2_ in the most hypoxic conditions remained higher than that for diving emperor penguins or exhausted race-horses, suggesting that the geese may not be at their aerobic limit. A surprising and unexpected finding was that venous blood temperature cooled during flight, which should enhance oxygen loading; this effect might be even more dramatic during flight at cold, high altitudes. And beyond the study of high-altitude flights, the latest results have implications for broader biological questions about the effects of these adaptations on animal distributions, the evolution of animals during periods with different oxygen levels, and even biomedical approaches to cardiorespiratory disease.
